# Rush Medical College

**Published:** 1847-08

**Authors:** 


### RUSH MEDICAL COLLEGE.

Four years have not yet elapsed since this institution, under the greatest difficulties, was organized in Chicago, then containing about eight thousand inhabitants. The project of a medical school in so small a town was regarded by many as chimerical and premature, because they had no conception of the prosperity and growth that awaited it. It was thought there could be no facilities for teaching practical anatomy and giving clinical instruction, which were in fact cogent reasons for doubting its success, as no school of medicine without them can properly teach these most important of all other branches of medical education.

But since, in the short space of time that has passed, the city has grown to number sixteen thousand inhabitants, and has established a hospital, (to which a lying-in department will be attached,) and a Dispensary; two charitable institutions for the treatment of the sick, to which students can have access, and in which regular instruction is given them by members of the faculty, it is conceded by all that Chicago is a most eligible site. And since the college has a commodious brick building, a library, extensive cabinets illustrating the several branches of medicine and the collateral sciences, a good set of chemical apparatus, &c., which have been procured by persevering energy on the part of the faculty, with ample means for clinical instruction and an abundant supply of material for dissection, no one can doubt its success. And as the school has grown regularly and rapidly, keeping pace with improvements around it, its classes increasing with the increase of the means of instruction—the first, in the winter of 1843–4, numbering twenty-two, and the last, in 1846–7, but three years intervening, being seventy students, it must be considered a healthy growth.

The members of the faculty, with whom our readers have become somewhat acquainted through the Journal, being devoted to the profession and each to his particular branch, and being wedded in interest and inclination to the college, give a guaranty of its future onward course and its wide extended usefulness. E.

